# Comparison of protein and energy supplementation to mineral supplementation on feeding behavior of grazing cattle during the rainy to the dry season transition

**DOI:** 10.1186/s40064-016-2603-9

**Published:** 2016-06-30

**Authors:** Rita Kelly Couto Brandão, Gleidson Giordano Pinto de Carvalho, Robério Rodrigues Silva, Daniel Lucas Santos Dias, Fabrício Bacelar Lima Mendes, Túlio Otávio Jardim D’Almeida Lins, George Abreu Filho, Sinvaldo Oliveira de Souza, Daniele Soares Barroso, Luana Marta de Almeida Rufino, Manuela Silva Libânio Tosto

**Affiliations:** Southwest Bahia State University–UESB, Itapetinga, Bahia Brazil; Federal University of Bahia-UFBA, Salvador, Bahia Brazil

**Keywords:** Bite, Efficiency, Pasture, Rumination

## Abstract

The aim of this study was to evaluate the effects of protein-energy or mineral supplementation on the ingestive behavior of dairy steers on pasture in the post-weaning phase during the rainy to dry season transition. Twenty-two ½ Holstein–Zebu dairy steers with an average initial body weight of 234 ± 16 kg were distributed into a completely randomized design into two groups: protein-energy supplementation and mineral supplementation offered *ad libitum*. The steers receiving protein-energy supplementation showed higher (P < 0.05) intake of dry matter (DM) and neutral detergent fiber (NDF) than those fed diets composed of mineral salt only. In addition, the animals that received protein-energy supplementation had longer period in grazing and spent on average more time per period eating at the trough (P < 0.05), however no significant differences were observed in the time per period in rumination and time per period in idle (P > 0.05). The supply of protein-energy supplement does not change the feeding behavior, except for an increase in the time spent feeding at the trough. The intake of protein-energy supplement improved the of DM and NDF feed efficiencies in grazing cattle during the rainy to the dry season transition.

## Background

Tropical pastures are considered the main component of the cattle production system in Brazil and their quality and availability affect directly the productivity of animals. However, production is not constant due to climatic variations which change forage availability throughout the year with quantitative and qualitative alterations in forage, especially during the dry period (Almeida et al. 2014). Thus, in the wet-dry transition period of the year, the forage quality is reduced because of the greater lignification of the plant components, with a decrease in the leaf:stem ratio, an increase in dead material and reduction of crude protein, which lead to lower digestibility of the forages.

The supplementation of grazing cattle consists of the act of supplying a source of additional nutrients to the system, which may generate changes in the forage intake, concentration of nutrients, availability of dietary energy, magnitude of the pool of biochemical precursors of the metabolism and animal performance (Paulino et al. 2004). The need for providing protein, energy and mineral supplementation to grazing cattle and the amounts that are supplied depend on the goals of the system and planned weight gain at the property on the quality and availability of pasture dry matter (Barbosa et al. 2008).

Moreover, rearing cattle on pasture is an activity characterized by factors linked to the environment, to the animal and to the pasture and their interactions may affect the search for food, in which the daily intake of forage is the key element for greater understanding of the feeding behavior (Palhano et al. 2007). Thus, the total forage intake by a grazing animal is the result of the accumulation of the forage consumed in each grazing action and of the frequency with which animals perform it during the time they spend feeding (Zanine et al. 2009). Through behavioral assessments, it is possible to evaluate the nutritional potential of diets and to adjust the animal feeding management aiming at better performance (Bastos et al. 2014).

The aim of this study was to evaluate the effect of protein-energy or mineral supplementation on the feeding behavior of dairy steers in their post-weaning phase on *Brachiaria brizantha* pastures during the wet–dry season transition.

## Methods

The experiment was conducted on Princesa do Mateiro Farm, located in Ribeirão do Largo, Bahia, Brazil. Twenty-two ½ crossbreed Holstein–Zebu steers with an average age of 10 months and an average initial body weight of 234.5 ± 16.0 kg were used. The total experiment lasted 84 days, consisting of three sub-periods of 28 days.

The steers were weighed, identified at the beginning of the experiment and were randomly divided into two groups. The animals were allocated in a completely randomized design, with two types of supplementation and 11 replicates. The following treatments were tested: 1—protein-energy supplementation 0.4 % of the body weight in supplement per day, balanced so as to meet the nutrient requirements for gain of 0,600 g day-1 (NRC 2001); and 2—mineral supplementation-mineral supplement offered *ad libitum*. The protein-energy supplement was supplied daily, at 1000h, in uncovered, collective plastic troughs with double access, and a linear dimension of 80 cm/animal (Tables 1 and 2).

**Table 1:** Proportion of ingredients of the supplements

Ingredient (%)	Protein-energy supplement	Mineral supplement
Corn	45.0	–
Soybean meal	45.4	–
Urea + AS^a^	5.0	–
Mineral mix^b^	4.6	100.0

^a^Urea + ammonium sulfate (9:1)

^b^Composition of the mineral mix: calcium 235 g; phosphorus 160 g; magnesium 16 g; sulfur 12 g; cobalt 150 mg; copper 1600 mg; iodine 190 mg; manganese 1400 mg; iron 1000 mg; selenium 32 mg; zinc 6000 mg; 1120 mg; fluorine (maximum) 1600 mg

**Table 2:** Chemical composition of the *Brachiaria brizantha* pasture and the protein-energy supplement

Item (g/kg DM)	*Brachiaria * *brizantha*	Protein-energy supplement
Dry matter (DM; in g/kg natural matter)	285.2	878.5
Organic matter	908.9	897.6
Crude protein	100.0	480.0
Ether extract	24.6	27.2
Neutral detergent fiber corrected for ash and protein	697.2	227
Acid detergent fiber	401.2	87.7
Total carbohydrates^a^	781.9	388.2
Non-fibrous carbohydrates^b^	155.4	158.4
Total digestible nutrients^c^	519.9	600.0

^a^Calculated by the equation proposed by Hall (2003)

^b^Calculated based on the equation proposed by Sniffen et al. (1992)

^c^Calculated based on the equation proposed by Weiss (1999)

The rotational stocking system was adopted in the pasture with *Brachiaria brizantha* cv. Marandu, in a area of  7.7 ha divided into six paddocks of equivalent areas. Thus, two paddocks were used per treatment and per experimental period. To avoid any effects from the pasture, animals from each treatment were rotated every 7 days across the paddocks.

The pasture was evaluated every 28 days (Table 3) and the availability of dry matter (DM) was determined following the method described by McMeniman (1997). The daily residual biomass (RB) was estimated according to the double-sampling method (Wilm et al. 1994). Before the cut, the DM of the biomass from the sample was estimated visually and the equation proposed by Gardner (1986) was utilized to calculate the forage biomass, expressed in kg/ha.

**Table 3:** Availability of dry matter (DM), residual biomass (RB), daily accumulation rate (DAR), forage allowance (FA), leaf:stem ratio and stocking rate of *Brachiaria brizantha* in the three experimental periods

Item	Period			Mean
	1st (March)	2nd (April)	3rd (may)	
Availability of DM (kg DM/ha)^a^	5285.0	4273.8	3859.8	4472.8
RB (kg DM/ha/day)^b^	179.1	152.6	137.8	156.5
DAR (kg DM/ha/day)^c^	20.2	18.3	13.6	17.3
FA (kg DM/100 kg body weight/day)^d^	15.6	8.5	5.9	10.0
Leaf:stem ratio	0.97	0.81	0.50	0.76
Stocking rate (animal unit/ha)^e^	0.88	0.99	1.02	0.96

^a^Determined as described by McMeniman (1997)

^b^Estimated according to the double-sampling method (Wilm et al. 1994)

^c^Determined by the equation proposed by Campbell (1966)

^d^Determined by the equation proposed by Prohmann et al. (2004)

^e^Considering the animal unit 450 kg body weight

The samples were collected, weighed and homogenized and composite samples were made for the separation of the forage components (leaf, stem and dead material). After the separation, each forage component was weighed to determine the morphological composition in percentage.

An external marker, titanium dioxide was utilized to estimate the concentrate intake. The marker was supplied at 10 g/animal day^−1^, mixed daily with the concentrate, for 7 days (Detmann et al. 2012). To estimate the voluntary intake of roughage, the indigestible neutral detergent fibre (iNDF) internal marker was used, obtained after 288 h of ruminal incubation (method INCT-CA F-009/1; Detmann et al. 2012). The forage samples collected by the simulated-grazing method were obtained according to Johnson (1978).

Forage samples were pre-dried in a forced-ventilation oven (55–60 °C) and ground in a Wiley mill to 1 and 2 mm sieve for laboratory analyses. Dry matter (DM; method INCT-CA G-003/1); organic matter (OM; method INCT-CA M-001/1); crude protein (CP; method INCT-CA N-001/1; ether extract (EE; method INCT-CA G-005/1); insoluble neutral detergent fiber corrected for ash and protein (NDFap): methods INCT-CA F-002/1, INCT-CA M-002/1 and INCT-CA N-004/1); and ADF (method INCT-CA F-004/1),contents were determined according to the techniques described by Detmann et al. (2012).

Non-fibrous carbohydrates corrected for ash and protein (NFCap) were determined by the equation recommended by Hall (2003). Total carbohydrates were calculated using the equation proposed by Sniffen et al. (1992) and total digestible nutrients (TDN), using the NDF corrected for ash and protein (Weiss 1999).

The evaluations of feeding behavior was evaluated in the 35th and 42nd day of the experimental period, with observations performed every 5 min, according to the method described by Silva et al. (2006), for a total period of 24 h per day by two observers trained, strategically placed so as not to disturb the animals. The following behavioral variables were observed: grazing time, rumination time, idle time and time spent feeding at the trough. The behavioral variables were considered mutually exclusive, as defined by Pardo et al. (2003). Time spent on feeding and rumination were calculated as a function of the intakes of DM and NDF (min/kg DM or NDF). The number of rumination chews and the time spent on ruminating each ruminal cud, for each animal, were obtained according to Burger et al. (2000) and the discretization of time series was performed as described by Silva et al. (2006).

The bite rate of the steers from each group was estimated as the time spent by the animal to perform 20 bites (Hodgson 1982). To calculate the bite mass, the daily intake was divided by the total number of daily bites (Jamieson and Hodgson 1979). The results of biting and swallowing observations were recorded in six occasions throughout the day, according to Baggio et al. (2009).

The variables g of DM and g of NDF per meal were obtained by dividing the average individual intake of each fraction by the number of feeding periods per day (in 24 h). The feed and rumination efficiencies, expressed in g DM/h and g NDF/h, were determined by dividing the average daily intake of DM and NDF by the total time spent feeding and/or ruminating in 24 h, respectively. The variables g of DM and NDF/cud were obtained by dividing the average individual intake of each fraction by the number of cuds ruminated per day (in 24 h).

The data were interpreted by analyses of variance, using the System for Statistical and Genetic Analyses (Sistema de Análises Estatísticas e Genéticas - SAEG; UFV, 2001), at 5 % probability level.

## Results

The steers that received protein-energy supplementation consumed more (P < 0.05) DM and NDFap than those fed only the mineral mix (Table 4). No significant differences were observed (P > 0.05) for the times spent on the grazing, idle and rumination activities, however the steers receiving protein-energy supplementation spent more time at the trough (P < 0.05) than the animals that received only mineral supplementation Additionally, no significant differences were observed (P > 0.05) for the total feeding and chewing times (Table 4).

**Table 4:** Intakes of dry matter (DM) and neutral detergent fiber corrected for ash and protein (NDFap), times spent on grazing, rumination, idle and feeding at the trough, total feeding and chewing times and feeding and rumination times as a function of DM and NDFap intakes by steers on pasture receiving protein-energy or mineral supplementation

Item^a^	Supplement		S.E.M^c^	P value
	Protein-energy	Mineral		
	kg/day			
DM intake	8.1	6.3	1.81	<0.001
NDFap intake	4.7	4.0	1.41	0.016

Item^a^	Supplement		S.E.M^c^	P value
	Protein-energy (Min/day)	Mineral		
Grazing^a^	618.9	653.7	14.19	0.107
Idle^a^	413.9	395.0	14.93	0.423
Rumination^a^	376.4	385.0	16.80	0.768
Trough^a^	30.9	6.2	7.79	<0.001
Total feeding time^a^	649.8	653.7	14.34	0.852
Total chewing time^a^	1026.1	1045.0	16.76	0.350

Item^a^	Supplement		S.E.M^c^	P value
	Protein-energy (Min/kg DM)	Mineral		
Feeding^b^	80.1	103.1	8.70	<0.001
Rumination^b^	46.4	60.7	8.37	0.044

Item^a^	Supplement		S.E.M^c^	P value
	Protein-energy (Min/kg NDFap)	Mineral		
Feeding^b^	138.8	164.2	10.63	0.004
Rumination^b^	80.4	96.7	10.71	0.129

^a^The behavioral variables were considered mutually exclusive, as defined by Pardo et al. (2003)

^b^Calculated as a function of the intakes of DM and NDF

^c^S.E.M = standard error of the mean

The times spent feeding (grazing + trough) in min per kg of DM and kg of NDFap were longer (P < 0.05) in the animals fed the mineral supplement as compared with those that received the protein-energy supplement, which may be due to the grazing time. In addition, the rumination time in min per kg of DM was shorter (P < 0.05) in the group that received protein-energy supplement compared with the group that received only mineral supplement, however, no differences were observed (P > 0.05) for the rumination time in min/kg NDFap (Table 4).

The animals receiving protein-energy supplementation showed higher bite rates and a lower number of bites per swallow (P < 0.05) compared with the animals fed mineral supplementation only. On the other hand, there were no significant differences (P > 0.05) between the types of supplement for bite mass, time per swallowed cud and number of bites per day (Table 5).

**Table 5:** Bite-related aspects of dairy steers receiving protein-energy or mineral supplementation on *Brachiaria brizantha* cv. Marandu pastures

Item	Supplement		S.E.M^d^	P value
	Protein/energy	Mineral		
Bite rate (number/s)^a^	0.7	0.5	0.68	0.012
Bite mass (g/DM)^b^	0.3	0.3	0.53	0.537
Bites per swallow (number)^c^	23.0	28.4	4.04	0.004
Time per cud swallowed (seconds)^c^	59.0	52.5	17.79	0.844
Bites per day (number)^c^	26,849	20,896	135.78	0.068

^a^Estimated as the time spent by the animal to perform 20 bites (Hodgson 1982)

^b^Daily intake divided by the total number of daily bites (Jamieson and Hodgson 1979)

^c^Were recorded in six occasions throughout the day according to Baggio et al. (2009)

^d^S.E.M = standard error of the mean

The steers that received protein-energy supplement showed a higher (P < 0.05) number of grazing and idle periods and periods feeding at the trough (Table 6). No significant differences were detected between the treatments for the number of rumination periods (P > 0.05). In addition, the animals that received protein-energy supplementation spent a shorter time per grazing period and a longer average time per period feeding at the trough (P < 0.05). No significant differences (P > 0.05) were observed for time spent per rumination period (Table 6).

**Table 6:** Number and time per period of feeding-related behavioral activities by dairy steers receiving protein-energy or mineral supplementation on *Brachiaria brizantha* cv. Marandu pastures

Item	Supplement		S.E.M.^c^	P value
	Protein-energy	Mineral		
Number of grazing periods^a^	13.4	10.1	3.58	0.027
Number of rumination periods^a^	15.4	13.8	2.92	0.088
Number of idle periods^a^	23.5	19.6	4.00	0.032
Number of periods feeding at the trough^a^	2.4	0.8	1.61	<0.001
Average time per grazing period (minutes)^b^	48.2	69.6	8.43	0.008
Average time per rumination period (minutes)^b^	24.5	28.4	4.83	0.120
Average time per idle period (minutes)^b^	18.2	20.9	4.72	0.246
Average time per trough period (minutes)^b^	13.6	7.3	3.56	<0.001

^a^Performed by counting periods of grazing, rumination and feeding in the trough (Silva et al. 2006)

^b^Obtained by dividing the daily times for each activity by the number of times the same activity (Smith et al. 2006)

^c^S.E.M = standard error of the mean

The intake in g DM/meal was not affected by the supplement (P > 0.05), however the intake in g NDF/meal was shorter in animals supplemented with protein-energy. The rumination efficiency in kg DM/h and the rumination in g DM/cud was higher (P < 0.05) in the animals consuming protein-energy supplement, furthermore the feed efficiency was higher (P < 0.05) in these animals (Table 7).

**Table 7:** Intakes of dry matter (DM) and neutral detergent fiber corrected for ash and protein (NDF), feed efficiency (kg DM and NDF/h) and rumination efficiency (kg DM and NDF/cud) of dairy crossbred steers on pasture receiving protein-energy or mineral supplementation

Item	Supplement		S.E.M^d^	P value
	Protein/energy	Mineral		
	Intake^a^			
g DM/meal	535.6	662.0	29.98	0.180
g NDF/meal	138.2	180.5	10.31	<0.001
	Feed efficiency^b^			
kg DM/h	0.80	0.58	0.897	0.023
kg NDF/h	0.46	0.36	0.534	0.001
	Rumination efficiency^b^			
kg DM/h	1.35	1.09	1.210	0.067
kg NDF/h	0.78	0.67	0.746	0.217
	Rumination^c^			
g DM/cud	17.53	14.13	3.612	0.012
g NDF/cud	10.16	8.76	2.995	0.111

^a^Obtained by dividing the average individual intake of each fraction by the number of feeding periods per day

^b^Obtained by dividing the average daily intake of DM and NDF by the total time spent feeding and/or ruminating in 24 h

^c^Obtained by dividing the average individual intake of each fraction by the number of cuds ruminated per day

^d^S.E.M. = standard error of the mean

## Discussion

In the animals consuming protein-energy supplementation, the association between the non-fibrous components and nitrogen compounds in the rumen provide the microrganisms with energy for microbial production and growth, improving the utilization of the fiber and increasing the digestibility of nutritional components. This allows for a higher forage intake, demonstrating a positive association effect between the supplement and the forage.

In the present study, the pasture showed a reduced leaf: stem ratio, which averaged 0.73 % (Table 3). In this situation, the animals tend to spend more time on grazing, seeking the most digestible parts of the plants. Additionally, because it is the most nutritive and palatable component of plants, the animals prefer the leaf blade; therefore, the animal decisions for the search for forage are preferentially based on the search for this component (Teixeira et al. 2010).

According Hodgson (1990), grazing times longer than 8–9 h per day, as observed in this study, may be indicative of limiting sward conditions to forage intake, because the stem density found in the pasture may act as a barrier to defoliation, reducing the ease at which the animal harvests the forage. This in turn promotes an increase in the duration of grazing, which may lead to a restriction of intake and the daily nutrient requirement not being met. Stivanin et al. (2014) evaluated the ingestive behavior of hoggets which received different types of supplement on ryegrass pasture, and observed that the daytime grazing is shorter when the hoggets receive supplements, regardless of the type of supplement.

Macedo Júnior et al. (2007) stated that effectiveness is the ability of a feedstuff or a diet to provide the motor and physical activity of the gastrointestinal tract, because ruminants retain fiber selectively in their rumen for the appropriate time for digestion. This occurs due to the consumption of long particles during feeding, which provide the necessary stimulation to trigger the rumination activity.

Because it has smaller and more digestible particles, the protein-energy supplement may reduce the rumination activity (Table 4) of the animals, which was demonstrated by Burger et al. (2000), who stated that concentrate and finely ground or pelleted hays reduce the rumination time, whereas roughages with a high cell wall content tend to increase it. The same way, Correia et al. (2015) evaluated feeding behavior of feedlot-finished young bulls fed diets containing peanut cake observed that the number of rumination periods increased with increasing dietary fiber content, reflecting the need for better processing of ruminal digesta to increase digestive efficiency.

The total availability of dry matter (4472.82 kg/ha; Table 3) was not limiting to intake; however, factors related to the animal, depending on the quality of the consumed forage, may limit the DM intake. The low leaf:stem ratio found in this study might have caused the lower bite rate by the animals that received mineral supplementation: 0.70 and 0.53 (n/s) for the protein-energy and mineral supplementations, respectively (Table 5). This lower bite rate observed for the animals that received only mineral supplementation may be explained by the fact that they spent more time selecting the most nutritive parts of the forage to meet their nutritional requirements, which increased the time spent on this activity and consequently the number of bites per swallow.

The bite rate is a measure that allows us to estimate how easily the forage is seized and, according to Hodgson (1985), the bite mass is the most important variable in the determination of the intake of grazing animals, affected by the structure of the forage sward. Almeida et al. (2014) evaluated the ingestive behavior of grazing heifers receiving crude glycerin supplementation during the dry–rainy season transition, observed that the addition of glycerin decreases and bite rate increases mass per bite in heifers supplemented during the dry–rainy season transition.

The highest number of grazing periods (Table 6), observed for the steers that received protein-energy supplementation in relation to those fed mineral supplement—13.36 and 10.12, respectively—evidence that the steers that consumed only mineral supplement had more intense grazing periods (TGP = 69.6 min), than the other groups (TGP = 48.2 min).

The longest average time per grazing period, observed in the group that received mineral supplement, can be explained by the greater selectivity during grazing, since the amount of dry matter and the availability of green leaves accessible on the pasture surface affect the time of permanence of ruminants in the search and harvest of food. Since the animal takes longer to stop grazing, due to the rumen fill (Trevisan et al. 2005), this led to a lower number of grazing periods (NGP) and consequently lower number of idle periods (NIP). Thus, this result is explained by the fact that the times in each period are the result of the total time spent on the activity division by the number of periods in which the activity.

The animals in the protein-energy supplement group received an additional uptake of nutrients originating from the supplement, their daily metabolic requirements were met faster than those of the animals that received only mineral supplement. According to Santana Júnior et al. (2013), the supply of concentrate reduces the time on search for forage and consequently the rumination time, thereby increasing the number of idle periods.

The higher intake of NDF (g/meal) by the steers that received mineral supplement (180.5 g) in relation to those that received protein supplement (138.2 g) was because the steers that received mineral supplement had only forage as food and when they grazed they did not consume only leaves, since the leaf:stem ratio was low (0.76 %), that caused them to consume stems and dead materials, which then increased the amount of ingested fiber. Diets with lower percentages of NDF provide a higher dry matter intake, requiring a shorter total feeding time per kg of DM by the animal, which indicates better feed and rumination efficiencies as a function of DM intake and according to Nicory et al. (2015) rumination efficiency is an important mechanism to evaluate the use of low-digestibility feeds.

## Conclusion

In general, the supply of a protein-energy supplement does not change the feeding behavior of the animals; however, it increases the time spent feeding at the trough. The consumption of protein-energy supplement during the wet–dry season transition improves the feed efficiencies of DM and NDF.
